# Towards the integration of antibiotic resistance gene mobility into environmental surveillance and risk assessment

**DOI:** 10.1038/s44259-025-00154-8

**Published:** 2025-09-16

**Authors:** Uli Klümper, Peiju Fang, Bing Li, Yu Xia, Dominic Frigon, Kerry A. Hamilton, Hunter Quon, Thomas U. Berendonk, Magali de la Cruz Barron

**Affiliations:** 1 Technische Universität Dresden, Institute of Hydrobiology, Dresden, Germany; 2 Tsinghua University, Tsinghua Shenzhen International Graduate School, Institute of Environment and Ecology, Shenzhen, China; 3 School of Environmental Science and Engineering, Southern University of Science and Technology, Shenzhen, China; 4 Civil Engineering, McGill University, Montreal, QC Canada; 5 The Biodesign Institute Center for Environmental Health Engineering, Arizona State University, Tempe, AZ USA; 6 School of Sustainable Engineering and the Built Environment, Arizona State University, Tempe, AZ USA

**Keywords:** Antimicrobial resistance, Environmental microbiology, Policy and public health in microbiology

## Abstract

Antibiotic resistance gene (ARG) mobility plays a crucial role in the spread of antimicrobial resistance across One Health settings. Current environmental surveillance often overlooks the significance of ARG mobility, limiting risk assessment accuracy. This perspective highlights that with recent methodological advances in detecting ARG mobility, relevant databases, and improved quantitative microbial risk assessment frameworks, the time to integrate ARG mobility into environmental antimicrobial resistance (AMR) surveillance and risk assessment is now.

## Antibiotic resistance gene mobility as a predictor of risk in environmental surveillance

The rise of bacterial antimicrobial resistance (AMR) poses a major threat to human and animal health^1^ with globally already 4.71 million deaths associated with and 1.14 million deaths directly attributable to bacterial AMR in 2021^2^. To mitigate the predicted rise in these numbers, it is important to assess the risks of AMR evolution, selection, and transmission within and across all interconnected One Health compartments (humans, animals, and the environment) to develop targeted intervention measures^3,4^. Assessing the risks connected with antimicrobial resistance genes (ARGs) in the human/clinical and the animal/veterinary sphere mainly relies on identifying and quantifying specific associations between ARGs and pathogenic host species in which AMR has led to worsened antibiotic treatment outcomes^5^.

When performing environmental AMR surveillance, where clinical treatment failure cannot be used as the main risk factor, translating environmental AMR surveillance data into quantitative risks remains a challenge^4,6–9^. This is because, unlike for chemical environmental pollutants, for which fate and transport modelling is more straightforward, complex microbial community behaviours regarding AMR spreading dynamics, such as horizontal gene transfer (HGT) of ARGs, across diverse environmental matrices remain challenging to predict^10,11^.

Approaches for multi-hazard risk assessments have been developed for chemicals, but these methods are more nascent for relating mixed microbial exposures to the probability of a specific direct effect^12^. Outbreaks of “classical” microbial pathogens (e.g., those resulting from a fecal-oral exposure), that cause immediate spikes in morbidity and mortality are more readily captured through disease surveillance and environmental detection, with risks defined through exposure probabilities and dose-response relationships^13^. In contrast to the aforementioned scenarios, AMR has been described as “the silent pandemic” because exposure to, or colonisation with, antimicrobial-resistant bacteria (ARBs) and various ARGs does not necessarily lead to immediate health consequences^14^. Instead, adverse outcomes typically only emerge following a subsequent infection and failure of clinical antibiotic treatment. Consequently, assessing the downstream risks associated with ARGs and ARBs during environmental surveillance depends not only on their abundance but also on additional risk factors, such as likelihood of exposure, human/animal host susceptibility, and potential for onward transmission.

## Current limitations in environmental AMR risk analysis

In a first attempt to assess which ARGs might be appropriate environmental surveillance targets as they pose particular epidemiological risks, Zhang et al.^15^ proposed four key indicators to rank individual ARGs: a) Circulation: Is the ARG shared between different One Health settings and does it seem increased in abundances due to human activities?; b) Mobility: Has the ARG been reported as encoded on a mobile genetic element (MGE) that increases its likelihood of transfer to a pathogen? c) Pathogenicity: Has the ARG been found in human or animal pathogens?; d) Clinical relevance: Has the ARG been related to worsened treatment outcome?. These four factors are reasonably easy to assess with currently available databases^15^, which allowed assigning risk ranks to individual ARGs. The abundance of high-risk ARGs can then be quantified through available surveillance methods including cultivation of ARB, individual ARG-based or high-throughput qPCR and metagenomics^16–19^. Furthermore, analysing the risk rank distribution in entire resistomes allows for assessing if an environment is moving towards a theoretically higher epidemiological AMR potential.

A crucial limitation of this method of AMR risk analysis is that it does not take the genetic and bacterial host context of the ARGs in the surveyed samples into account, but rather assesses risk based on worst-case historical genetic contexts^15^. In particular, an ARG that has once been found in a pathogen and on a mobile genetic element that was connected to clinical treatment failure will always be ranked as a high potential risk (Risk Rank I). This is true even if an eventual epidemiological linkage is not apparent in the environmental sample where the ARG might appear chromosomally in a non-pathogenic, indigenous bacterium that can neither colonize humans nor animals, or perhaps does not have a high degree of transmissibility to pathogenic microorganisms that can go on to infect human hosts or meaningfully affect other ecological processes. The resulting overestimation of the potential epidemiological AMR risk in certain environments could subsequently lead to flawed prioritization and mitigation measure selection. This would result in setbacks in combatting the spread of AMR. Thus, it remains necessary to evaluate the relationships between environmental surveillance data and clinical outcomes rather than relying on qualitative postulation of potential risks. This should involve not only considering ARG abundance and potential risk ranks, but also quantitatively assessing ARG-host associations, ARG mobility, and any associated adverse outcomes. We define ARG mobility here as the association of an ARG with an MGE that facilitates HGT between microbial cells of the same or different species. The association with plasmids as the main drivers of ARG transfer is particularly important^20^, as plasmids have been shown to facilitate ARG transfer at high rates across phylogenetically diverse bacterial host species^21–23^, hence increasing the risk of ARGs ending up in human or animal pathogens.

To determine the realized quantitative risks, mobility and bacterial host information need to be integrated into quantitative microbial risk assessment (QMRA) or other modelling frameworks as in some cases observing direct linkages with cases and/or outbreaks is challenging. QMRA frameworks include hazard identification, exposure assessment, dose-response analysis, and risk characterization^13^ to quantify and better understand microbial health risks in defined scenarios or applications. They are often used to evaluate posed risks against established benchmarks or regulations^24^ and inform management and policy decisions; furthermore, integrating techniques for addressing the complexities of AMR has been identified as a top QMRA research need^25^.

When prioritizing the integration of ARG-host or ARG-MGE associations into QMRAs for clinical and veterinary surveillance, priority should be given to ARG-host associations, as ARGs appearing in human or animal pathogens can lead to immediate clinical treatment failure, hence risk. Contrary, in environmental surveillance, it might be preferable to prioritise the integration of ARG-MGE associations: ARG-host associations might be relevant in direct and/or short time-course exposure scenarios of humans or animals in the environment. In the environment, ARGs often persist across extended time frames^26^ and may undergo multiple bacterial host transitions before reaching a pathogenic host capable of infecting humans or animals^27^. In such complex and often poorly traceable systems, the association of ARGs with MGEs, here defined as ARG mobility, represents a useful proxy for future dissemination potential. While the direct clinical impact of any single environmental ARG may be rare or difficult to quantify, mobility increases the likelihood of horizontal transfer, persistence, and eventual uptake by relevant pathogens. We acknowledge that clear outbreak-level linkages between environmental ARGs and disease outcomes remain limited^28^; nevertheless, they have been documented^29^. However, we hypothesise that environmentally modulated AMR transmission occurs more frequently than currently detected, and that the environment may play a larger, though undercharacterised, role in shaping clinical AMR risks. In this context, risk models typically still focus not on all ARGs or all hosts and MGEs, but on clinically relevant ARG-MGE or ARG-MGE-bacteria combinations with plausible links to human or animal health as endpoint measures^13,25^. These may be defined based on epidemiological data (e.g., treatment failure, WHO-priority pathogens^30^), known resistance phenotypes, ARG-MGE linkage, or bioinformatic evidence of ARG-MGE-host associations in pathogens, as most environmental ARG hosts will usually be non-pathogenic. Thus, understanding ARG mobility offers a tractable and pragmatic entry point for identifying high-risk scenarios and moving from unpredictable toward more predictable environmental AMR dynamics.

## Methodological advances to quantify ARG mobility

### The current AMR surveillance toolbox

Quantitatively evaluating ARG mobility and its subsequent integration into QMRA has been a challenge to date. The main environmental AMR surveillance methods currently applied have various limitations when it comes to capturing the mobility potential of ARGs. qPCR techniques have a high sensitivity (with detection limits around 1 gene copy per 10^5^ to 10^7^ genomes) but do not allow further characterization of the diversity of ARG sequences or their associations with MGEs and bacterial hosts. They are further limited to individual targets^7^. Metagenomic approaches, on the other hand, can detect a large number of ARGs and MGEs in small subsamples of microbiomes^31,32^, but have limited sensitivity (with detection limits around 1 gene copy per 10^3^ genomes) and generally focus on simple correlation analysis between ARGs and MGEs or relative abundances, leading to limited insights into the ARG mobility potential. Other methods to investigate ARG-MGE associations include the exogenous capture of plasmids^33,34^, inverse PCR^35,36^, or epicPCR^37^, but these suffer from very low throughput and nontrivial analyst training requirements, incompatible with broad-based surveillance.

Despite this already well-stuffed molecular toolbox, none of these techniques can alone fulfil all the needs of an adequate One Health surveillance designed for risk assessment due to their sensitivity, information, and complexity limitations. Consequently, we propose that such a surveillance system should be constructed with multiple tiers. The first tier is to achieve sensitive and sufficient information to recognize dissemination patterns of ARGs, yet simple enough to allow scale-up to large numbers of labs and samples. The later tiers could then be increasingly informative on the genomic context of ARGs (association with MGEs and carrying populations) despite being less sensitive and more complex in their implementation. This approach would provide an optimal design between the cost of implementation and data needs.

**Fig. 1:**
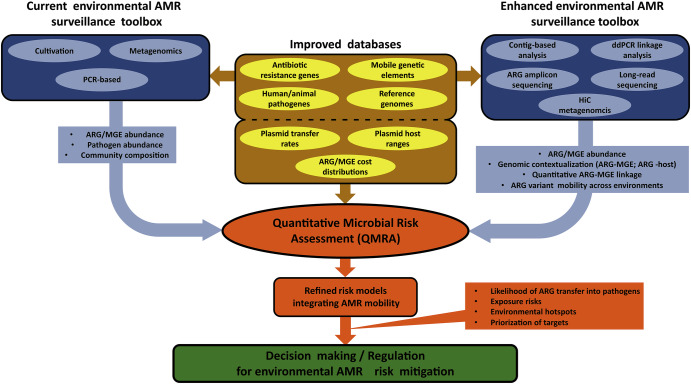
Conceptual overview of how current and enhanced environmental AMR surveillance methods contribute to risk modelling and mitigation via integration into quantitative microbial risk assessment (QMRA). The “Current toolbox” (left) illustrates common surveillance approaches used to quantify ARG or pathogen abundances, including PCR-based methods, cultivation, and short-read metagenomics. The “Enhanced toolbox” (right) includes emerging techniques that improve the resolution of ARG mobility and ARG host analysis. These methods vary in analytical sensitivity, throughput, and the granularity of information they provide, ranging from simple quantification (e.g., qPCR) to different degrees of genomic contextualization and mobility inference with high specificity and sensitivity (e.g. ddPCR linkage analysis) or broad screening and low sensitivity (e.g., contig-based analysis & long-read sequencing. Together, these approaches can be implemented in a tiered framework: low-cost, high-throughput tools support broad surveillance, while high-resolution methods are applied to priority samples or locations to estimate key parameters such as ARG mobility potential, plasmid host range, and fitness cost distributions. Together with the ongoing improvement of databases, this integration of different methods enables more refined QMRAs that incorporate ARG dissemination likelihoods and exposure risks, informing prioritisation and decision-making for AMR mitigation.

Recent methodological advances can be considered in designing these new surveillance systems that go beyond only quantification and consider an increasing number of One Health reservoirs (Fig. 1). Such advances, reviewed in detail below, include amplicon and whole-community metagenome sequencing-based methods, PCR-based genotype association assays, improved bioinformatic pipelines that allow contig-based analysis or the integration of long-read-based sequencing into environmental AMR surveillance and improved databases. The novel approaches developed below are reaching the quantitative and qualitative information to characterize ARGs and their observable mobility at the level necessary for their efficient integration in QMRAs. Rather than proposing a fixed or exhaustive classification, we view these surveillance methodologies as existing along a gradient of analytical resolution, sensitivity, and implementation complexity. Depending on the surveillance and risk assessment goals as well as resource constraints, different combinations of methods may be applied to balance throughput with contextual insight. This framing avoids prescriptive tiers in a rapidly evolving AMR surveillance toolbox while preserving the core idea of integrating complementary tools to achieve risk-relevant resolution.

Although this manuscript focuses on including mobility-related indicators of risk for known ARGs into QMRA, future work should also consider the inclusion of complementary surveillance strategies for detecting mobilisation of novel ARG variants^38^. Recent studies have identified potential ‘evolution hubs’ in the environment that may support the emergence of new resistance traits^39–41^. Target-agnostic approaches, including functional metagenomics^42^ and culture-enriched sequencing^43^, will be important for monitoring these hotspots and expanding the surveillance landscape beyond known resistance determinants.

### Assessing ARG sequence type mobility across different environments through targeted amplicon sequencing

Adequate ARG surveillance requires the sensitive detection of ARGs down to the sequence variant in different reservoirs to elucidate circulation and mobility across surveillance transects. This could ultimately reveal spread and survival dynamics of ARG variants between interconnected source and sink environments. For this purpose, a novel and multiplexable targeted amplicon sequencing approach to detect sequence diversity of ARGs down to the single nucleotide polymorphism level and hence determine ARG variant mobility has recently been developed^44,45^. Here, up to 20 ARGs and MGEs are simultaneously amplified using multiplex PCR and barcoded for sample pooling. By pooling multiplexed PCR products from several reactions of the same sample, several 100 s of ARGs or MGEs can be sequenced in a single run, which enables fast and low-cost analysis of sequence diversity of multiple targets. The feasibility of the method was demonstrated in a study of 16 wastewater treatment facilities in Quebec that indicated the presence of *bla*_OXA_-type ARGs at all locations^45^. However, sequence variant analysis revealed that among the detected *bla*_OXA_ variants, those related to *bla*_OXA-2_ and *bla*_OXA-10_ were among the most widespread across locations. These variants have been previously reported in association with MGEs and observed in multiple bacterial hosts^46,47^, although their transferability patterns across environments remain to be fully characterised. Hence, this method allows for a high-resolution assessment of which ARG sequence variants are indeed transferred between different interconnected surveillance settings and thus of high-risk nature, presenting crucial information for QMRA.

### Quantification of ARG-plasmid linkage through multiplexed digital droplet PCR

Digital PCR-based methods have been widely applied for rapid, easy-to-use, and cost-efficient surveillance during the COVID-19 pandemic^48^ and provide equal opportunities in future AMR surveillance. Recently a quantitative assay taking advantage of multiplexed digital droplet PCR (ddPCR) technology to simultaneously assess the abundance of ARGs and MGEs in combination with their physical linkage on the same DNA fragment has been validated^49^. ddPCR is characterized by the random distribution of individual DNA molecules from an environmental sample across thousands of droplets in which PCR takes place^50^. Individual targets can be simultaneously quantified using different fluorochromes. By statistically evaluating the proportion of droplets in which ARG and MGE marker genes co-occur, it becomes possible to infer the percentage of detected ARG copies in direct physical linkage with a specific plasmid or even classes of plasmids, other MGEs or bacterial hosts depending on the choice of primers^49^. With recent ddPCR machines being able to multiplex up to 12 targets, this method can easily be upscaled to quantify the linkage between an ARG and the majority of plasmid classes present within a sample, to assess the overall mobility potential of an ARG. As validated ddPCR primer sets and probes to amplify diverse ARGs^51^ and plasmid groups^52,53^ are already widely available, such multiplexing can be straightforwardly achieved for targeted environmental surveillance operations. Due to the rapid nature of ddPCR assays, quantitative results regarding the association of a specific or even multiple ARGs with different groups of plasmids could hence be gained within less than a day after sampling to feed into QMRA or other models.

### Quantification of ARG mobility through advanced bioinformatic analysis of short-read metagenomic sequencing

The analysis of short-read metagenomic sequencing to survey ARGs in environmental samples has been utilized since the early 2010s^54^. Ongoing advances in bioinformatic analysis methods and tools have significantly enhanced our ability to not only identify and quantify ARGs from metagenomic datasets, but also determine their mobility. Assembly-based tools such as metaSPAdes and MEGAHIT allow the construction of long contigs and even the reconstruction of entire genomes or plasmids from short reads^55,56^. If an ARG is located on the same contig as bacterial host-specific taxonomic markers or chromosomal genes, this can provide evidence of ARG-host linkage, while co-location of an ARG on a contig with MGE marker genes indicates ARG mobility. By predicting open reading frames (ORFs) (through e.g., Prodigal), ARG-carrying contigs can be identified through, for example, alignment to CARD and SARG databases using DIAMOND^57,58^. In addition to existing reference databases for ARGs (e.g., CARD, SARG), several curated databases (e.g., MobileOG^59^, MobileGeneticElementDatabase^60^) have been developed to support MGE identification and annotation. Through advancements in the identification and characterization of MGEs (including plasmids, integrative conjugative elements (ICEs), integrons, and transposons) using ever-improving databases, mobile ARG carrying contigs can be identified. Identification tools based on plasmid databases, such as PlasmidFinder and Platon can identify plasmid sequences^61,62^, whereas specialized analysis tools such as PlasmidSPAdes^63^ further enable the differentiation of plasmid-derived contigs already during assembly. For ICE detection, specialized software (e.g., ICEfinder) can identify integrase genes and conjugation machinery components within assembled contigs^64^. IntegronFinder can identify integrons in bacterial genomes, predict their genetic structure, including integrase genes and associated gene cassettes, and classify them as mobile or sedentary based on their genomic context^65^. By taking the coverage of contigs into account, mobile ARGs can not only be identified but also quantified. The applicability of such advanced bioinformatic mobility analysis for surveillance purposes was for example demonstrated in the Shenzhen Bay megacity basin, where sulphonamide ARGs *sul*1 and *sul*2 and beta-lactam ARGs *bla*_KPC-2_, *bla*_TEM-1_, *bla*_GES-2_, and *bla*_GES-5_ were identified as particularly mobile^66^. Additional sensitivity regarding the detection of rare, mobile ARGs during surveillance can be achieved through selective enrichment of plasmidic DNA from environmental samples ahead of metagenomic sequencing^67–69^. However, it is important to note that enrichment techniques inherently alter the original abundance distributions of ARGs and MGEs, thereby limiting the quantitative interpretation of resulting data. Whereas such approaches improve sensitivity for detecting low-prevalence mobile ARGs, they preclude the use of results for absolute abundance estimation, a key requirement in exposure-based risk assessments such as QMRA. Consequently, enrichment-based data should be interpreted qualitatively or semi-quantitatively and may be more appropriate for hazard identification or the prioritisation of ARGs and MGEs for further targeted quantification. Although these enrichment strategies enhance the likelihood of capturing rare, mobile ARGs and their flanking regions, they do so at the expense of preserving native community structure and abundance. Therefore, the resulting data are best suited for qualitative analyses, such as identifying high-risk ARGs, characterising mobility contexts, or informing model structure in QMRA rather than for exposure quantification.

Recent evaluations have also highlighted serious limitations to exclusively relying on short-read based approaches for mobility analysis. Short-read assemblies often break at conserved ARG regions due to their presence across multiple genomic contexts, leading to fragmented contigs and disrupted linkage information^70^. These challenges are even more pronounced for plasmids and integrative elements, which often remain poorly assembled or undetected in complex microbiomes. Furthermore, the above mentioned post-assembly identification tools such as PlasmidFinder or Platon have shown limited precision and accuracy in distinguishing plasmid-derived contigs from chromosomal sequences in metagenomic datasets^71^. Additionally, the assembly process itself leads to a loss of sequence diversity and sensitivity, with reduced recovery of rare or fragmented ARG/MGE variants. Hence, short-read sequencing remains a cost-effective and widely available approach to obtain ARG and MGE abundance data for subsequent QMRA, whereas reliance on it alone for ARG mobility assessment is currently insufficient and should be considered a methodological limitation. Hybrid sequencing strategies, which integrate the depth and accuracy of short reads with the contextual resolution of long reads (discussed below), are likely required to balance sensitivity, specificity, and practical implementation for surveillance and risk assessment purposes.

### Implementation of long-read metagenomic sequencing to quantify the mobility of ARGs

In contrast to the assembly of short reads into long enough fragments to infer the genomic context of ARGs and their bacterial hosts, long-read metagenomic sequencing offers a straightforward solution for the simultaneous detection of the ARG-carrying populations and mobility of ARGs. Currently, the most widely used long-read platforms are Oxford Nanopore and PacBio sequencing. Whereas the HiFi reads generated by PacBio can achieve a Q30 quality, equivalent to metagenomic assembled contigs^72^, Oxford Nanopore produces lower quality but longer reads that are particularly useful for revealing ARG genomic contexts. Similar to short-read annotation, long reads also require the alignment to reference databases for ARG and MGE detection (see above). However, since the reads tend to have lower per base accuracy, for instance, 99% accuracy for R10 chemistry in Nanopore sequencing, raw reads often require correction or protein-based annotation strategies to address frameshift and basecall errors before annotation (extensively reviewed elsewhere^73^). Alternatively, alignment-based tools such as NanoARG^74^ apply DNA-DNA comparison to sidestep translation errors, and methods such as the IDEEL test^75^ allow estimation of frameshift rates in long-read assemblies.

The potential of Nanopore-based metagenomic profiling was demonstrated using wastewater microbiomes where it revealed high proportions of plasmid-carried, mobile ARGs^76^. In addition to revealing ARG-MGE associations, long reads can span the surrounding regions flanking ARGs encoded on chromosomes, thus allowing the assembly of multiple long reads and enhancing the identification of the bacterial hosts of ARGs at the species level. However, for environmental samples with high diversity, it often remains challenging to achieve sufficient coverage for effective long-read-based assembly of genomes of ARG-carrying bacteria, which usually display low prevalence. However, recent advances in adaptive sampling, such as the metaRUpore protocol, designed to enrich rare species in metagenomes, could provide future promising solutions to this limitation^77^. By simultaneously providing qualitative ARG mobility and ARG bacterial host information, long-read sequencing approaches can be valuable for feeding into QMRAs. Still, because long reads generate fewer total reads per sample, estimating the relative abundance of ARGs or quantifying their mobility is inherently more difficult. Increasing sequencing depth can mitigate some variability^78^, but often correction strategies such as mapping short-read abundance profiles to the long-read contigs are required. Additionally, most bioinformatics tools designed for metagenomic quantification (e.g., Bracken) were originally optimized for short-read data, making direct abundance estimation from long reads less straightforward^79^. Moreover, long-read libraries regularly suffer from uneven fragment size distributions, which can bias abundance estimates and limit the reliability of long-read data alone for quantification^80^. Thus, to generate the quantitative information needed for QMRA, hybrid approaches integrating short-read sequencing for precise abundance estimation and long-read sequencing for contextualization may still be necessary.

### Utilisation of Hi-C sequencing for ARG- and MGE-host association analysis

Another promising approach to resolving ARG-host and MGE-host associations is chromosome conformation capture sequencing (Hi-C), which captures physical proximity between DNA molecules within intact microbial cells^81^. This technique enables the linkage of ARGs, plasmids and other MGEs to their specific bacterial hosts in metagenomic samples^82,83^. When combined with hybrid assemblies using short-read (e.g., Illumina) and high-fidelity long-read sequencing (e.g., PacBio HiFi), Hi-C has been shown to generate lineage-resolved metagenome-assembled genomes (MAGs) alongside their associated extrachromosomal elements, thereby facilitating high-resolution profiling of ARG-host-MGE relationships^72^. These capabilities make Hi-C a valuable tool for understanding ARG mobility in complex microbiomes and for supporting more refined integration of mobility data into risk models such as QMRA. However, the method currently faces significant limitations in the context of routine surveillance. These include relatively low throughput, high costs, and the need for complex and non-standardised bioinformatic workflows, which limit scalability and broader implementation. Although Hi-C holds great promise for detailed investigations or targeted validation studies, its application in high-volume, policy-relevant environmental surveillance remains constrained at this stage. As sequencing costs decline and workflows become more standardised, Hi-C-based methods represent, however, a promising future addition to the environmental AMR surveillance toolbox.

### Incorporation of viability and internal standards into ARG mobility analysis

Although current surveillance methods provide information on ARG abundance and potential mobility, they cannot generally distinguish between viable and non-viable bacterial hosts and do not incorporate standardised normalisation across samples, limiting their ability to infer actual transfer risk. To overcome this, recent developments in environmental analytical microbiology have introduced internal cellular standards to enhance absolute quantification and viability assessments in metagenomic analyses^84^. These internal references allow for normalisation of sequencing data and the distinction between viable and non-viable cells, adding a biologically relevant dimension to the interpretation of ARG abundance and distribution. The metabolic activity of host cells is a key factor in horizontal gene transfer potential, and methods that allow assessing viability can therefore improve the risk relevance of detected ARGs. Moreover, internal standards can serve to validate the multiplexed ddPCR and amplicon-based sequencing methods mentioned above by anchoring quantitative results to a consistent cellular baseline. The feasibility of integrating such data into risk frameworks has already been demonstrated in QMRA applications assessing ARG exposure in recreational water settings^85^. These approaches offer promising complementary tools for improving the accuracy and interpretability of ARG mobility analyses during environmental AMR surveillance.

## Integrating ARG mobility data into quantitative microbial risk assessment for AMR

QMRA^13^ is a general modelling framework that has been applied across diverse sectors, including food safety^86^, environmental exposure to pathogens^87^ and healthcare settings^88^. Its applicability in environmental AMR surveillance depends not on the specific environmental matrix, but on the availability of hazard, exposure, and dose-response data relevant to the system under study^25^. ARG mobility data offer an opportunity to refine risk prioritisation within these models. For instance, such data could help address key questions including: (i) which ARG-MGE combinations present the highest likelihood of horizontal transfer into pathogens; (ii) which environmental matrices, such as wastewater effluents, biosolids, or irrigation water, pose the greatest risk and therefore warrant prioritization in monitoring and mitigation; and (iii) what target concentrations of mobile ARGs or ARBs in a given matrix would represent a threshold of public health concern. The ability of QMRAs to answer these types of questions will be greatest in data-rich contexts, where environmental surveillance and human exposure pathways are relatively well characterised (e.g., reclaimed water reuse), while more data-limited settings will require additional empirical inputs to reduce uncertainty in model outputs.

The relationship between AMR surveillance and QMRA is mutually reinforcing; enhancing and centralizing detection methods and data availability improves risk assessments, which in turn helps identify key areas for more effective data collection, surveillance, and management as well as methodological gaps to target with future technological development. Traditional applications of QMRA have relied on pathogen concentrations and exposure scenarios, often overlooking the dynamic nature of AMR dissemination, particularly through ARG mobility. Whereas some recent QMRA studies have assessed AMR-related risks, many have not accounted for ARG mobility and HGT^89–91^. Njage et al.^92^ incorporated HGT in QMRA for extended-spectrum beta-lactamase (ESBL) *E. coli* in lettuce, using proportion or frequency estimates to assess overall HGT within the risk assessment framework. Two QMRA studies that integrated HGT rates, one for ESBL *E. coli* in recreational water^27^ and another for methicillin-resistant *Staphylococcus aureus* (MRSA) in treated wastewater^93^, found that HGT had minimal impact on final risk estimates. But, they could play a larger role when considering host in vivo kinetics and impact on final doses of resistant pathogens to which humans or animals are ultimately exposed. This was largely because HGT had minimal impact on the final risk estimates in the modelled scenarios, due to the inherently low HGT rates of these pathogens under environmental conditions, with risk estimates instead being dominated by environmental factors such as dilution. However, these assumptions rely on quantitative data on ARG mobility for proper model integration, which is limited across different scales between the environment and the human body^94^.

### Remaining data needs and future prospects for integrating ARG mobility into QMRAs

With the methods above, quantifying which proportion of ARGs in an environmental sample are mobile on which types of MGEs has become possible. Key data gaps for validating QMRA models of AMR that incorporate HGT in complex microbiomes require in-depth knowledge regarding the transfer rates and bacterial host ranges of different types and classes of MGEs to estimate their subsequent spread, as well as the costs of these MGEs to evaluate their loss dynamics. Whereas many of these ecological processes are inherently context-dependent, shaped by microbial physiology, environmental conditions, and community interactions, they do not need to be completely mechanistically resolved to be useful in QMRA. Empirical data on ARG or ARB persistence within specific environments can serve as proxies for these dynamics, enabling their integration into risk modelling frameworks in a tractable and scenario-specific manner. Still, research into estimating these factors for plasmids is advancing rapidly, while for other MGEs early estimates are available, but more targeted studies are needed.

Regarding plasmid transfer rates, a recent literature review by Quon et al.^95^ compiled conjugation rates from environmental, clinical, and agricultural sources, quantifying and summarising them for use with QMRA. Their findings revealed rates spanning over 12 orders of magnitude, with the highest rates in bacteria sourced from fish and municipal wastewater. However, limited time-series information currently available for evaluating the kinetics of HGT processes makes extrapolation of these processes to relevant dissemination and exposure pathways difficult. For integrating plasmid and thus ARG loss dynamics, a recent study compiled estimates of the distribution of plasmid costs, based on a systematic review of available empirical data^96^. Regarding bacterial hosts, recent machine learning-based methods now allow predicting the host ranges of, at least, plasmids (as the most relevant MGEs in AMR spread) based on their genetic composition^97–99^. By comparing these host ranges to the database of known human pathogens^100^ pathways of transfer into pathogens can be identified. Together with the available information regarding conjugation rates, loss dynamics, and pathogen abundances in different environments, QMRAs or other models could then estimate the likelihood of, at least, plasmid-encoded ARGs ending up in pathogens as a risk endpoint. For other MGE types, such as integrative conjugative elements^101^ or transposons^102^, or other HGT mechanisms such as transformation^103,104^, transduction^105,106^ or vesicle transfer^107,108^ early estimates of transfer rates or host ranges are available. However, the available data is currently too scarce to integrate them into QMRAs, highlighting the need for future research.

It remains uncertain how ARG mobility can impact human dose-response and disease progression after exposure to resistant pathogens^109^. Quon et al.^95^ and Moralez et al.^94^ emphasize the importance of scale, setting, and proper unit selection for better analysis and integration with established models, such as for the estimation of population dynamics risks^27,110^. A significant limitation for human dose-response modelling of ARB and accounting for ARG mobility is the availability of in vivo experiments and kinetics or in vitro reactor-based trials as proxies^13,111^.

Additional methods have been proposed to improve AMR risk estimation, particularly through the use of whole-genome sequencing (WGS) for increased accuracy in strain identification^112^. Omics-based approaches have also been developed to support risk prioritisation and potential integration into absolute risk frameworks such as QMRA. For example, MetaCompare enables relative risk ranking of resistome profiles and could inform downstream quantitative risk models^113^. Other methods focus on ranking mobile ARG risks and assessing bacterial host-associated viability to identify ARGs with higher dissemination potential^15,85^. Lastly, narrowing the set of pathogenic genera considered during hazard identification has been proposed^114^, and this approach could be extended to include ARG-host dimensions relevant to QMRA. A combination of targeted and non-targeted approaches could ultimately contribute to identifying the most informative ARG, host strain and MGE targets that should be monitored as proxies for AMR risk assessment during environmental surveillance^115^.

Overall, improved data availability and accuracy across regions and scales will improve QMRA for AMR through better identification and measurement techniques, improved source tracking, better quantification of ARG mobility (HGT). Ultimately this will lead to refined modelling approaches for risk estimation during environmental AMR surveillance. However, the interpretability and credibility of model outputs also depend on the ability to validate risk estimates. In most cases, full validation against epidemiological endpoints, such as infection rates or clinical outcomes, remains infeasible due to limited availability of corresponding data for environmental AMR exposure pathways^25^. Instead, model performance is often assessed by evaluating individual components (e.g., exposure concentrations, decay kinetics, or mobility parameters) against experimental or field-derived measurements, while accounting for associated uncertainties. As more empirical and longitudinal surveillance data become available, especially those linking environmental ARGs to clinical cases, the development of structured validation frameworks will become increasingly feasible. In the interim, transparency in model assumptions, sensitivity analyses, and iterative refinement remain essential for enhancing confidence in the application of QMRA to AMR risk assessment contexts^25^.

## Conclusion

The development of novel molecular methods and advances in metagenomic sequencing and bioinformatic analysis to determine ARG-MGE associations allows to accurate determination of ARG mobility as a prominent risk indicator during environmental AMR surveillance. Furthermore, currently available QMRA models for environmental AMR have already demonstrated that ARG mobility-related data can be integrated to transform surveillance results into measurable risks in defined scenarios^27,92,93^. With the improvement of databases regarding ARGs, MGEs, conjugation rates, plasmid loss rates, and human pathogens, we hence argue that now is the time to integrate ARG mobility into QMRA for environmental AMR. This effort needs interdisciplinary collaborations, including policy-making, to ensure that the insights gained are translated into effective regulatory measures and enhanced global monitoring networks that align with One Health principles.

## Data Availability

No datasets were generated or analysed during the current study.
